# Prognostic potential of preoperative circulating tumor cells to predict the early progression recurrence in hepatocellular carcinoma patients after hepatectomy

**DOI:** 10.1186/s12885-023-11629-0

**Published:** 2023-11-27

**Authors:** Zhan Lu, Hanghang Ni, Xihua Yang, Lihao Tan, Haixiao Zhuang, Yunning Mo, Xingyu Wei, Lunan Qi, Bangde Xiang

**Affiliations:** 1 Department of Hepatobiliary Surgery, Guangxi Medical University Cancer Hospital, 71# Hedi Road, Qingxiu District, Nanning, Guangxi 530021 People’s Republic of China; 2 Key Laboratory of Early Prevention and Treatment for Regional High-Frequency Tumors, Ministry of Education, Nanning, People’s Republic of China; 3 Guangxi Medical University, Nanning, People’s Republic of China; 4 Department of Surgical Oncology, Chenzhou No. 1 People’s Hospital, Chenzhou, People’s Republic of China; 5 Guangxi Liver Cancer Diagnosis and Treatment Engineering and Technology Research Center, Nanning, People’s Republic of China

**Keywords:** Hepatocellular carcinoma, Circulating tumor cells, Hepatectomy, Early progression recurrence

## Abstract

**Background:**

The role of circulating tumor cells (CTCs) in prognosis prediction has been actively studied in hepatocellular carcinoma (HCC) patients. However, their efficiency in accurately predicting early progression recurrence (EPR) is unclear. This study aimed to investigate the clinical potential of preoperative CTCs to predict EPR in HCC patients after hepatectomy.

**Methods:**

One hundred forty-five HCC patients, whose preoperative CTCs were detected, were enrolled. Based on the recurrence times and types, the patients were divided into four groups, including early oligo-recurrence (EOR), EPR, late oligo-recurrence (LOR), and late progression recurrence (LPR).

**Results:**

Among the 145 patients, 133 (91.7%) patients had a postoperative recurrence, including 51 EOR, 42 EPR, 39 LOR, and 1 LPR patient. Kaplan–Meier survival curve analysis indicated that the HCC patients with EPR had the worst OS. There were significant differences in the total-CTCs (T-CTCs) and CTCs subtypes count between the EPR group with EOR and LOR groups. Cox regression analysis indicated that the T-CTC count of > 5/5 mL, the presence of microvascular invasion (MVI) and satellite nodules were the independent risk factors for EPR. The efficiency of T-CTCs was superior as compared to those of the other indicators in predicting EPR. Moreover, the combined model demonstrated a markedly superior area under the curve (AUC).

**Conclusions:**

The HCC patients with EPR had the worst OS. The preoperative CTCs was served as a prognostic indicator of EPR for HCC patients. The combined models, including T-CTCs, MVI, and satellite nodules, had the best performance to predict EPR after hepatectomy.

**Supplementary Information:**

The online version contains supplementary material available at 10.1186/s12885-023-11629-0.

## Introduction

Hepatocellular carcinoma (HCC) is the third leading cause of cancer-related deaths worldwide [1]. Surgical treatment has the most favorable outcomes for the early- and medium-stage HCC. The currently available surgical treatments for HCC patients include hepatectomy and liver transplantation (LT) [2–4]. Radical hepatectomy is the most commonly used surgical treatment for HCC because the donors for LT are limited, and only a small subset of available patients meet the Milan criteria. However, the 5-year recurrence and metastasis rates are still as high as 50–70% after surgery [5, 6].

Numerous studies have shown that both the HCC recurrence time and pattern of tumor recurrence are important factors, affecting the prognosis of HCC patients [7, 8]. Moreover, the selection of a post-recurrence treatment option is also based on the pattern of HCC recurrence [9, 10]. A previous study [9] has divided recurrence into four types: Type I, a single recurrent tumor in the liver; Type II, number of recurrent tumors in the liver is > 1 and ≤ 5; Type III, vascular invasion and/or extrahepatic metastases, such as lung, bone, lymph node, brain, etc. with ≤ 5 intrahepatic tumors; and Type IV, > 5 recurrent nodules in the liver with or without vascular invasion and extrahepatic metastasis. Type III and IV are the postoperative extrahepatic metastasis and intrahepatic dissemination, respectively, and are progression recurrence (PR). Most PR-developing patients have refractory recurrences and usually poor treatment outcomes [9]. The patients with Type I and II and a few Type III HCC recurrences can also benefit from ablation, surgery, intervention, targeted therapy, immunotherapy, and other treatments [11, 12]. Some patients can even achieve the same effects as those of the first resection through re-surgical resection or salvage LT [13–15]. However, postoperative PR is often fatal with an over 50% death rate within a year [9]. PR is correlated with tumor dissemination, which usually appears within 2 years. Therefore, studying the patients with early PR (EPR) might reveal more distinct clinical and biological features of these patients. However, fewer studies have focused on this issue.

Early and mid-stage HCC patients also suffer from EPR. Primitive surgery might not improve the survival outcomes, which might even get worse. EPR might be related to preoperative micrometastases and tumor cell dissemination. Studies have focused on the role of circulating tumor cells (CTCs) in the metastasis of malignancies in recent years [16, 17]. The detection of CTCs in the peripheral blood of patients with solid tumors indicates the metastasis of malignancy; its assessment using clinicopathological indicators alone is difficult [18, 19]. The major route of HCC metastasis is the hematogenous route. Previous studies have also shown differences in the numbers and types of CTCs in different recurrence subtypes [17, 20]. Not surprisingly, patients with advanced-stage cancer and high tumor burden have increased numbers of CTCs in their blood. However, EPR also occurs in early- and mid-stage HCC, which might also be related to the clinical characteristics of patients [21, 22]. Therefore, once the high-risk patients are identified, the postoperative adjuvant treatments, such as adjuvant transcatheter arterial chemoembolization (a-TACE), Lenvatinib, Sorafenib, etc., can be planned, and the follow-up frequency might be increased [9, 11].

After hepatectomy, EPR in HCC patients indicated a severely poor prognosis; however, its risk factors have not been explored yet. Moreover, the correlation between CTCs and EPR has also not been studied. This study analyzed the data from HCC patients performed a preoperative CTC analysis and a long-term follow-up to explore the correlations between CTCs and EPR, followed by exploring the clinical risk of EPR.

## Materials and methods

### Patient enrollment

A total of 145 HCC patients, who underwent radical surgical resection in the Tumor Hospital of Guangxi Medical University, Nanning, Guangxi Province, China, between 2014 and 2019, were enrolled in this retrospective study. The inclusion criteria for the recruitment of patients were as follows: 1) the HCC patients treated with curative hepatectomy, which was defined as the complete macroscopic removal of tumor tissues, resection margin negative, and no detectable intra- or extra-hepatic metastatic lesions remaining; 2) the patients having no other malignancies; 3) the patients who did not undergo any prior anti-tumor treatment; 4) the patients with the liver function of Child-Pugh class A or B; 5) the patients whose complete clinicopathological and follow-up data were available. Moreover, the patients, who died of postoperative complications or underwent non-radical resection, were excluded.

We calculate the sample sizes at http://powerandsamplesize.com/Calculators/ for this time-to-event analysis. The sample size calculation was based on (1) level of significance: 2-sided test at α = 0.05; (2) power (1 –β): 80%; (3) effect size: Hazard Ratios (HRs, θ) of ≥ 3.0 are considered clinically significan; (4) θ_0_: the hazard ratio hypothesized under the null hypothesis is considered to be 1; pE: the overall probability of the event occurring within the study period is taken to be 0.3; pA: proportion of sample in group was adopted as 0.3, following previous studies [9].

Clinical and laboratory characteristics of all the patients, including age, sex, hepatitis B surface antigen (HBSAg), hepatitis B virus DNA level (HBV-DNA), body mass index (BMI), total bilirubin (TBil), serum alpha-fetoprotein (AFP) level, Child-Pugh grade, tumor size, tumor number, Edmondson’s grade, resection margin, microvascular invasion (MVI), liver cirrhosis, and ki-67 levels, etc., were collected. The HCC stage was evaluated according to the Barcelona Clinic Liver Cancer (BCLC) staging classification [23], and the degree of tumor differentiation was defined according to the Edmondson grading system [24]. The postoperative adjuvant therapy included adjuvant transarterial chemoembolization (A-TACE), incisal margin radiotherapy, adjuvant targeted therapy, and immunotherapy. Risk factors for postoperative recurrence included Tumor size ≥ 5 cm, Histological grade ≥ 3, and the presence of MVI or satellite nodules. The recurrence typing was based on the recently developed four-class classification by Qi et al. [9]. The protocol for this study was approved by the ethics committee of the Tumor Hospital of Guangxi Medical University, Nanning, Guangxi Province, China. All the participants provided written informed consent.

### Isolation and detection of CTCs

The CTC analysis was performed 1–2 days preoperatively using the CanPatrol CTC enrichment system and in situ hybridization (ISH) technique. The peripheral blood samples (5 mL, anticoagulated with ethylenediaminetetraacetic (EDTA)) were collected after discarding the first 2 mL of blood. The red blood cells were removed using a red blood cell lysis buffer and resuspended in phosphate-buffered saline (PBS), containing 4% formaldehyde (Sigma, St. Louis, MO, USA) for 5 min before filtration.

CTCs were isolated using the CanPatrol CTC enrichment system with a filtration tube, containing a membrane with 8-μm diameter pores (Sur Exam, Guangzhou, China), a manifold vacuum plate having a valve setting (SurExam, Guangzhou, China), an E-Z96 vacuum manifold (Omega, Norcross, GA, USA), and a vacuum pump (Auto Science, Tianjin, China). RNA-ISH was to identify and examine the expression levels of epithelial and mesenchymal genes in CTCs using three types of nucleic acid probes. The detected target sequences included CD45 (leukocyte biomarker), EpCAM, CK8/18/19 (epithelial biomarkers), vimentin, and twist (mesenchymal biomarkers). Using the expression levels of these genes, the types of CTCs were identified, including mesenchymal-CTCs (M-CTCs), epithelial-CTCs (E-CTCs), and hybrid CTCs (E/M-CTCs). The hybrid CTCs included the fluorescence of both epithelial and mesenchymal genes.

### Follow-up and recurrence

The postoperative follow-up of patients was performed every 1–2 months for 1 year and then every 3 months thereafter until the occurrence of recurrence. The follow-up programs included liver function, AFP, and at least one contrast imaging scan, such as contrast-enhanced computed tomography (CT), magnetic resonance imaging (MRI), ultrasonography, etc. The post-recurrence treatments included surgery, radiofrequency ablation (RFA), TACE, targeted therapy, immunotherapy, etc. Then, the follow-up after recurrence was performed according to the clinical schedules until the patient’s death, and the patient’s treatment-related deaths were recorded.

Recurrence was assessed based on the combined analysis of new lesions in the residual liver and other body parts revealed using contrast-enhanced scanning, the patient’s past medical history, AFP level, and even pathological biopsy. The time of recurrence-free survival was calculated, starting from the day of surgery to the diagnosis of recurrence or death, while overall survival (OS) time was calculated, starting from the day of surgery to the death of a patient or last follow-up. The endpoint of the follow-up for all the patients was August 10, 2022, or until the patient’s death. Recurrences, occurring within 2 years were considered early recurrence (ER), while those occurring after 2 years were considered late recurrence (LR).

### Statistical analyses

The clinical and pathological features of all the enrolled patients underwent binary classification and were expressed as n (%). The cut-off values of CTCs were derived from the optimal cut-off values of receiver operator characteristic (ROC) curves corresponding to the maximum value of the Jörden exponent. For the remaining indicators, the common clinical cut-off values were used as references. The CTCs and their subtypes were not normally distributed and were compared using Mann–Whitney test. The predictive performance of different indicators was evaluated using the area under the ROC curve (AUC). Kaplan–Meier survival curve analysis was performed to compare the recurrence-free survival (RFS) and OS between groups. Univariate and multivariate Cox proportional hazards regression models were used to analyze the correlations between patient characteristics and EPR. A *P*-value of < 0.05 was considered statistically significant. All the statistical analyses were performed using IBM SPSS Statistics 25 and GraphPad Prism 8.

## Results

### Characteristics of the study population

A total of 145 patients, including 124 males (85.5%) and 21 (14.5%) females, with early and intermediate HCC, whose complete data were available, were included in this study, and no surgery-related deaths occurred. The basic clinical features of the patients are listed in Table 1. Among the 145 patients, 131 patients had HBV-related HCC, 101 patients had tumors ≥ 5 cm, 84 patients had MVI, and 41 patients indicated the presence of satellite nodules. Moreover, 40.7% of patients exhibited pathologic liver cirrhosis.

**Table 1:** Baseline characteristics of the included 145 patients with hepatocellular carcinoma in the study

Characteristic	Value
Patients, n	145
Sex (male)	124 (85.5%)
Age, year (≥ 45)	83 (57.2%)
BMI (≥ 24 kg/m^2^)	52 (35.9%)
Diabetes mellitus	12 (8.3%)
HBsAg (positive)	131(90.3%)
HBV-DNA(≥ 5 × 10^2^ IU/mL)	101 (69.7%)
Child-Pugh class (A stage)	137 (94.5%)
TBil (≥ 17.1 μmol/L)	48 (33.1%)
ALB (≥ 35 mg/L)	127 (87.6%)
ALBI (> -2.60)	79 (54.5%)
ALT (≥ 40 U/L)	67 (46.2%)
AST (≥ 40 U/L)	78 (53.8%)
PT (≥ 13 s)	72 (49.7%)
INR (≥ 1)	111 (76.6%)
Platelet count (≥ 225 × 10^9^/L)	67 (46.2%)
AFP (≥ 200 μg/mL)	81 (55.9%)
Tumor size (≥ 5 cm)	101 (69.6%)
Multiple lesions (yes)	41 (28.3%)
Inflow blood occlusion (yes)	109 (75.2%)
Tumor capsule (complete)	114 (78.6%)
Resection margin (≥ 1 cm)	45 (31.0%)
MVI (positive)	84 (57.9%)
Histological grade (≥ 3)	77 (53.1%)
Histological cirrhosis (yes)	59 (40.7%)
Satellite nodule (yes)	41 (28.3%)
Ki67 (≥ 35%)	63 (43.4%)
Postoperative adjuvant therapy (yes)	62 (42.8%)
T-CTC count (> 5/5 mL)	77 (53.1%)
E-CTC count (> 1/5 mL)	81 (55.9%)
M-CTC count (> 2/5 mL)	30 (20.7%)
M/E-CTC count (> 3/5 mL)	46 (31.7%)

Values are shown as n (%)

*Abbreviations*: *AFP* Alpha-fetoprotein, *ALB* Albumin, *ALBI* Albumin-bilirubin, *ALT* Alanine aminotransferase, *AST* Aspartate aminotransferase, *BMI* Body mass index, *E-CTCs* Epithelial-circulating tumor cells, *HBV-DNA* Hepatitis B virus DNA, *HBsAg* Hepatitis B surface antigen, *HCC* Hepatocellular carcinoma, *INR* International normalized ratio, *M-CTCs* Mesenchymal-circulating tumor cells, *M/E-CTCs* Mesenchymal/epithelial-circulating tumor cells, *MVI* Microvascular invasion, *PT* Prothrombin time, *TBil* Total bilirubin, *T-CTCs* Total-circulating tumor cells

In total, 138 of the included patients were with high risk factors of recurrence and 62 of them performed postoperative adjuvant therapy. Among the 62 patients, EOR occurred in 28 (45.2%), EPR in 16 (25.8%), and LOR in 18 (29.0%). Of the patients with high-risk recurrence factors who did not receive postoperative adjuvant therapy, EOR occurred in 23 (30.3%), EPR in 26 (34.2%), LOR in 26 (34.2%), and LPR in one (1.3%). In both groups, there was no significant difference in EOR (*p* = 0.071), EPR (*p* = 0.286), and LOR (*p* = 0.516).

According to the BCLC staging, 104 patients were in stages 0-A, and 41 patients were in stage B. There were significant differences in the distribution of T-CTCs and subtypes between patients in stages 0-A and B (Fig. S1).

### Differences between T-CTCs and subtypes in ER and LR

Among the 145 patients, 12 patients had no recurrence, while 133 patients experienced recurrence, among which, 93 patients had ER, while 40 patients had LR. The patients without recurrence or death were followed for more than 36 months with the longest follow-up time of 101 months.

**Fig. 1:**
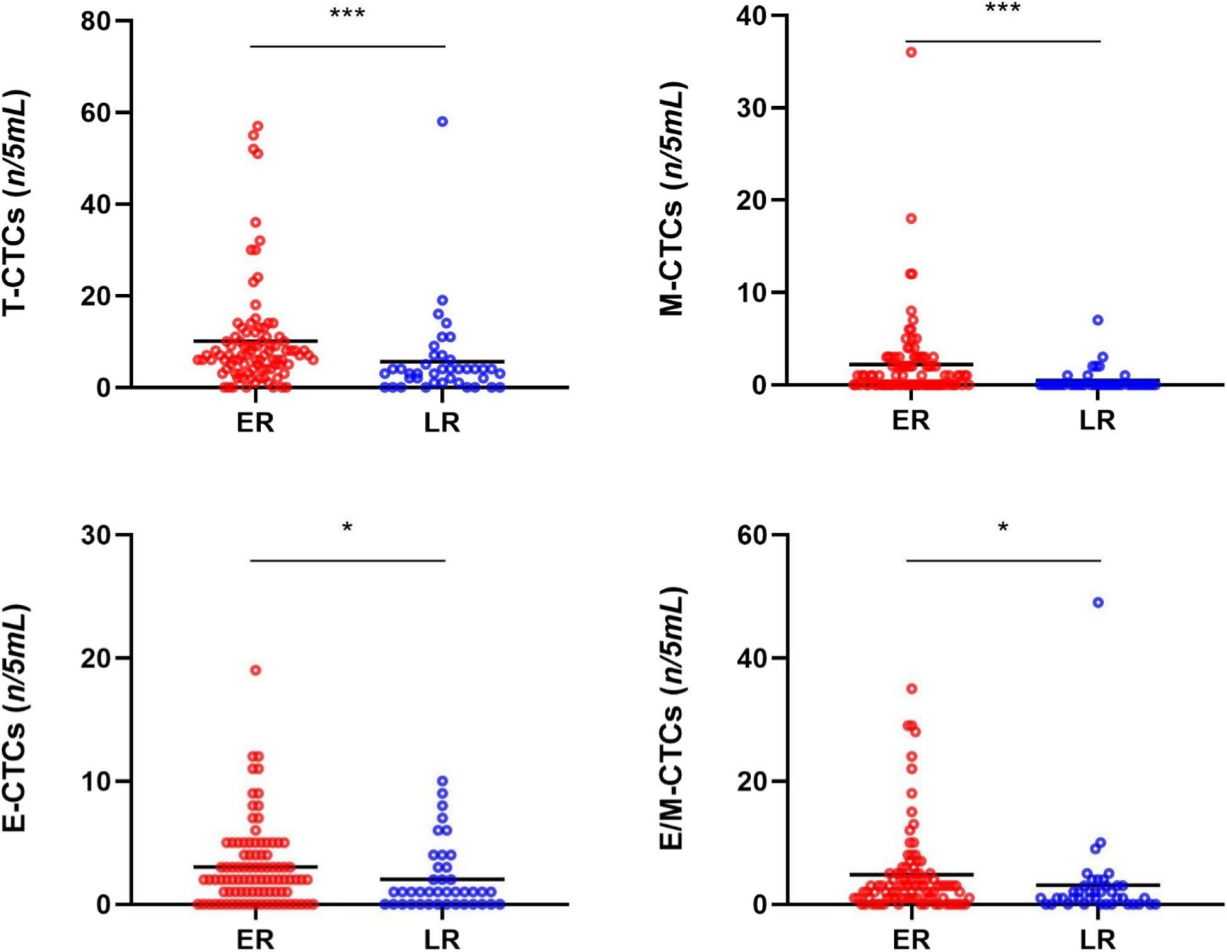
Distribution of T-CTCs and subtypes in the HCC patients with ER and LR

The median counts of T-CTCs in the ER and LR groups were 7 per 5 mL (7/5 mL) *vs.* 3/5 mL. 1/5 mL *vs.* 0 in M-CTCs, 2/5 mL *vs.* 1/5 mL in E-CTCs, and 3/5 mL *vs.* 1/5 mL in E/M-CTCs. Mann–Whitney test showed significant differences in the number of T-CTCs (*P* < 0.01), M-CTCs (*P* < 0.01), E-CTCs (*P* < 0.05), and E/M-CTCs (*P* < 0.05) between the two groups (Fig. 1).

The ROC curve analysis was performed to identify the efficacy and cut-off values of T-CTCs and their subtype for the prediction of ER and LR. T-CTCs (0.707) showed the highest AUC as compared to M-CTCs (0.697), E-CTCs (0.617), and E/M-CTCs (0.635). The best cut-off value of T-CTCs count for predicting ER was > 4/5 mL (Fig. S2).

### Correlations between CTCs and EPR

**Fig. 2:**
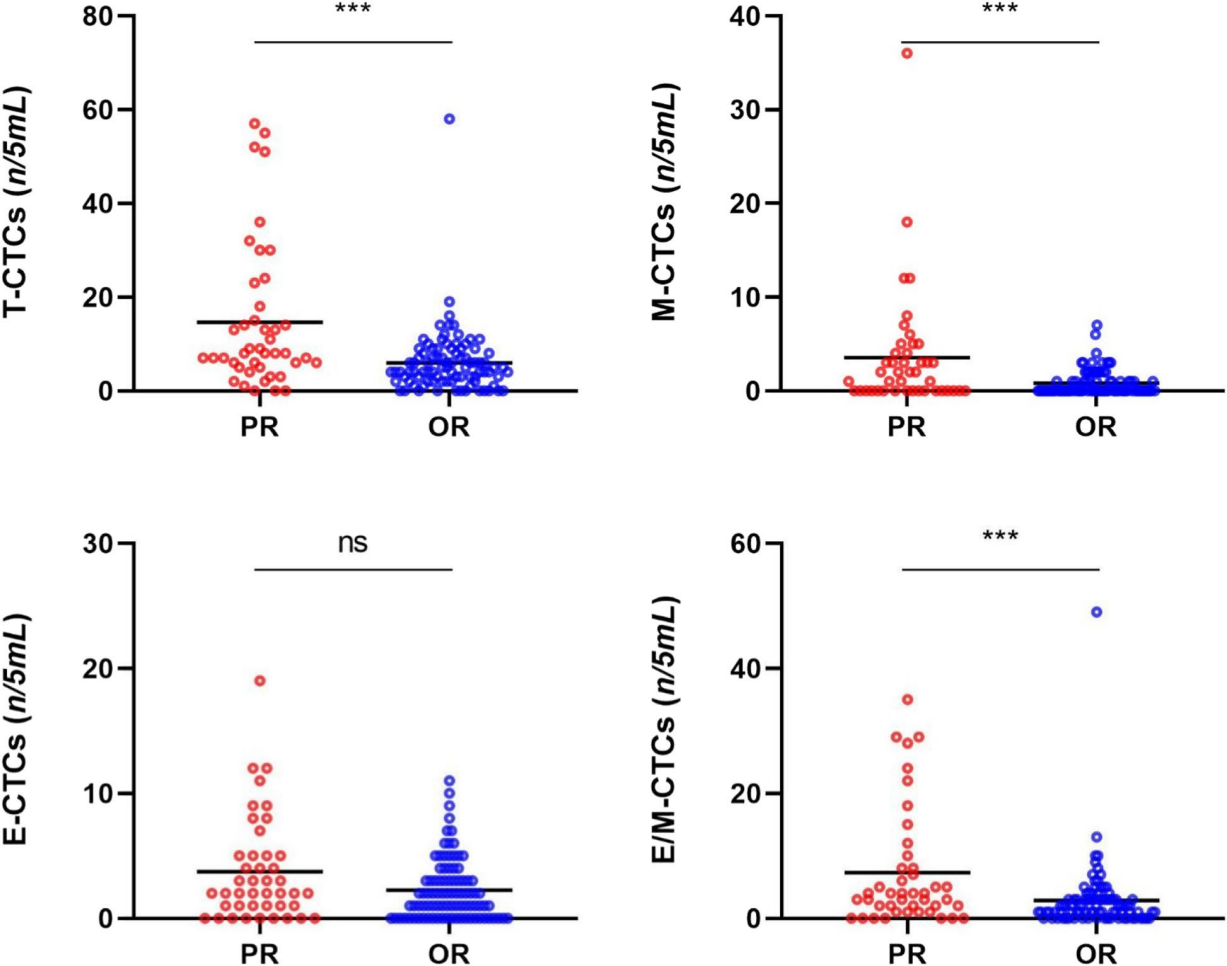
Distribution of T-CTCs and subtypes in the HCC patients with PR and OR

Among the 133 patients with recurrence, 90 patients developed oligo-recurrence (OR), and 43 patients suffered PR. Comparing the PR and OR groups showed that except for E-CTCs, there were significant differences in the T-CTCs, M-, and E/M-CTCs (*P* < 0.05) between the two groups. The median counts of T-CTCs between the PR and OR groups were 8/5 mL *vs.* 4/5 mL, 2/5 mL *vs.* 0 in M-CTCs, 2/5 mL *vs.* 1/5 mL in E-CTCs, and 4/5 mL *vs.* 1/5 mL in E/M-CTCs (Fig. 2).

**Fig. 3:**
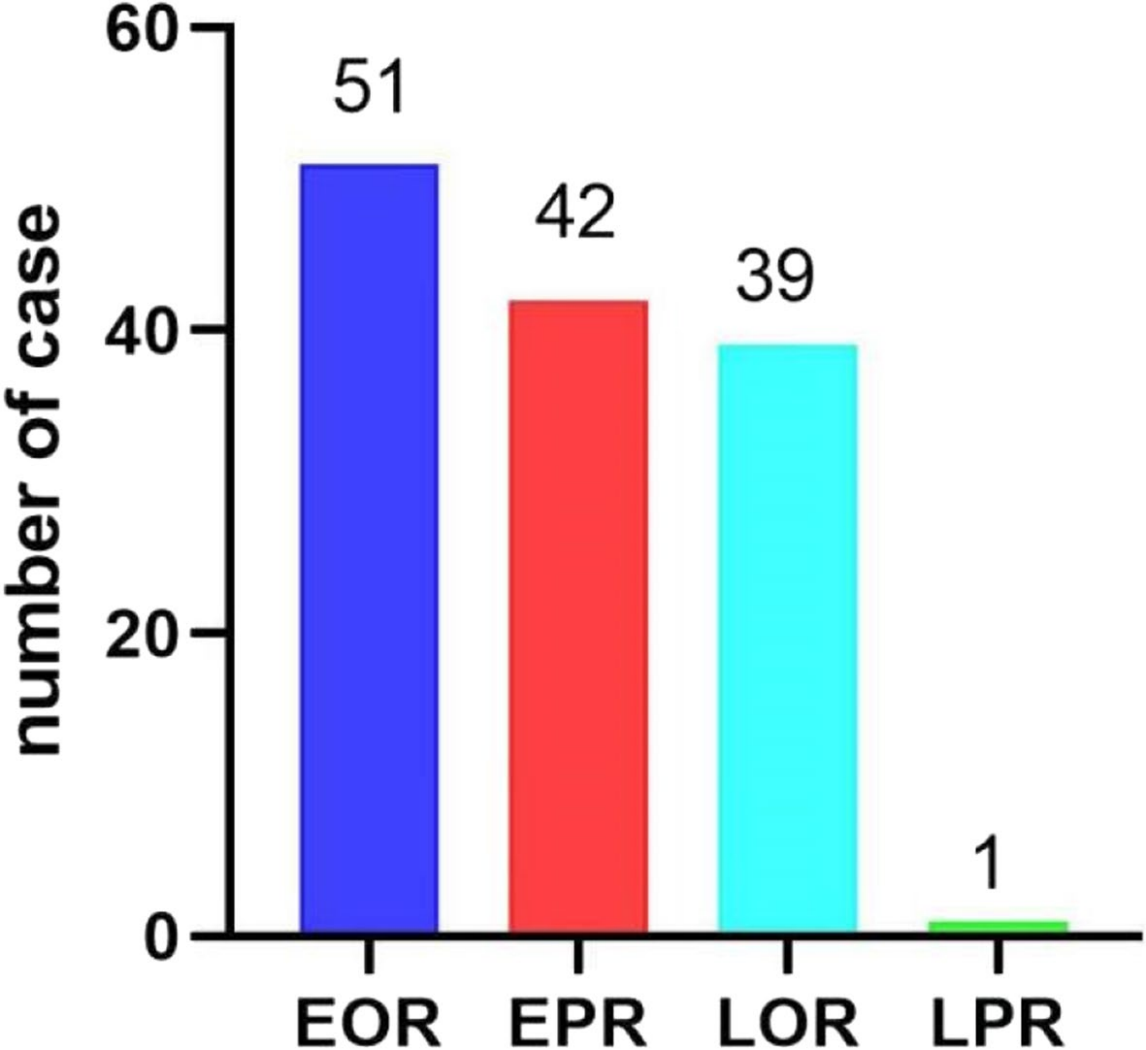
Distribution of HCC patients with the four types of recurrences

As shown in Fig. 3, among the 133 patients with recurrence, 51 patients and early OR (EOR), 42 patients had EPR, 39 patients had late OR (LOR), and only 1 patient had late PR (LPR).

**Fig. 4:**
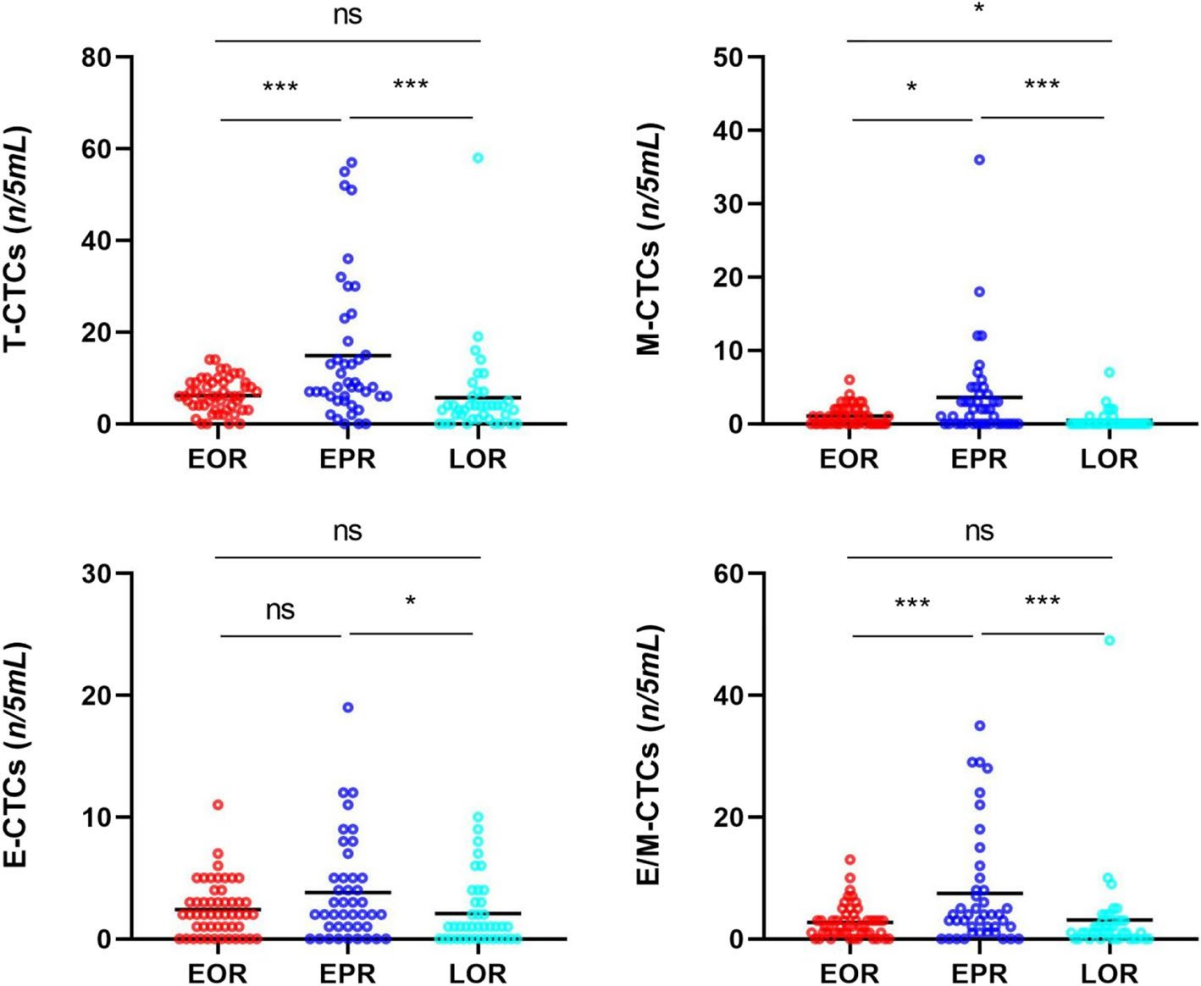
Distribution of T-CTCs and subtypes in the HCC patients with EOR, EPR, and LOR

The CTC comparative analysis of all the patients in the EOR, EPR, and LOR groups and a few patients in the LPR group was performed. The results showed that the counts of T-CTCs, M-CTCs, E-CTCs, and E/M-CTCs in the EPR group were significantly higher as compared to those in the EOR and LOR groups (*P* < 0.05), except for E-CTC counts between the EPR and EOR groups (*P* > 0.05). However, there were no significant differences in the counts of T-CTCs, E-CTCs, and E/M-CTCs between the EOR and LOR groups, and only a slight difference was observed in the M-CTC count (Fig. 4).

**Fig. 5:**
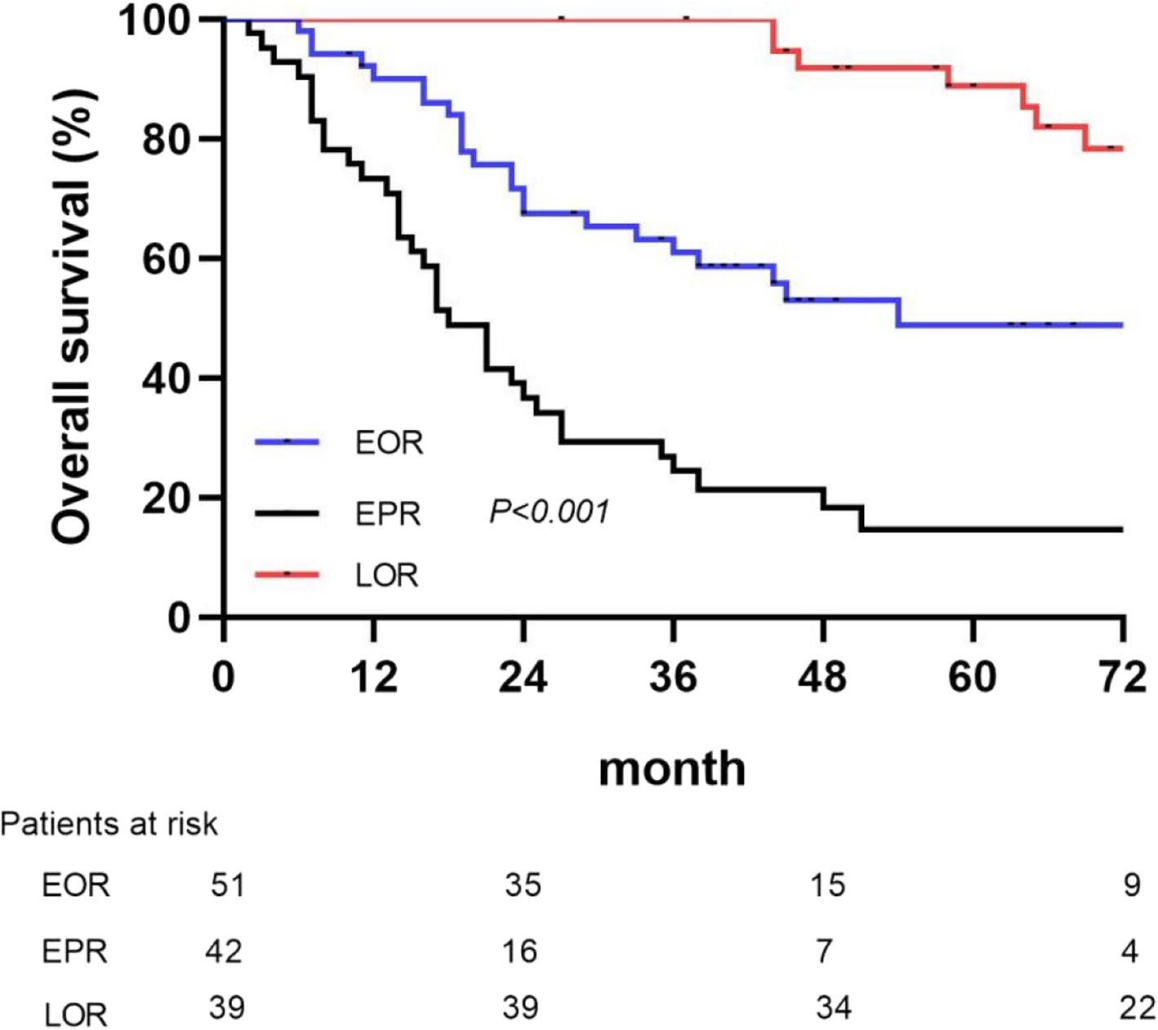
Kaplan–Meier survival curve analysis of OS in the HCC patients with EOR, EPR, and LOR

**Fig. 6:**
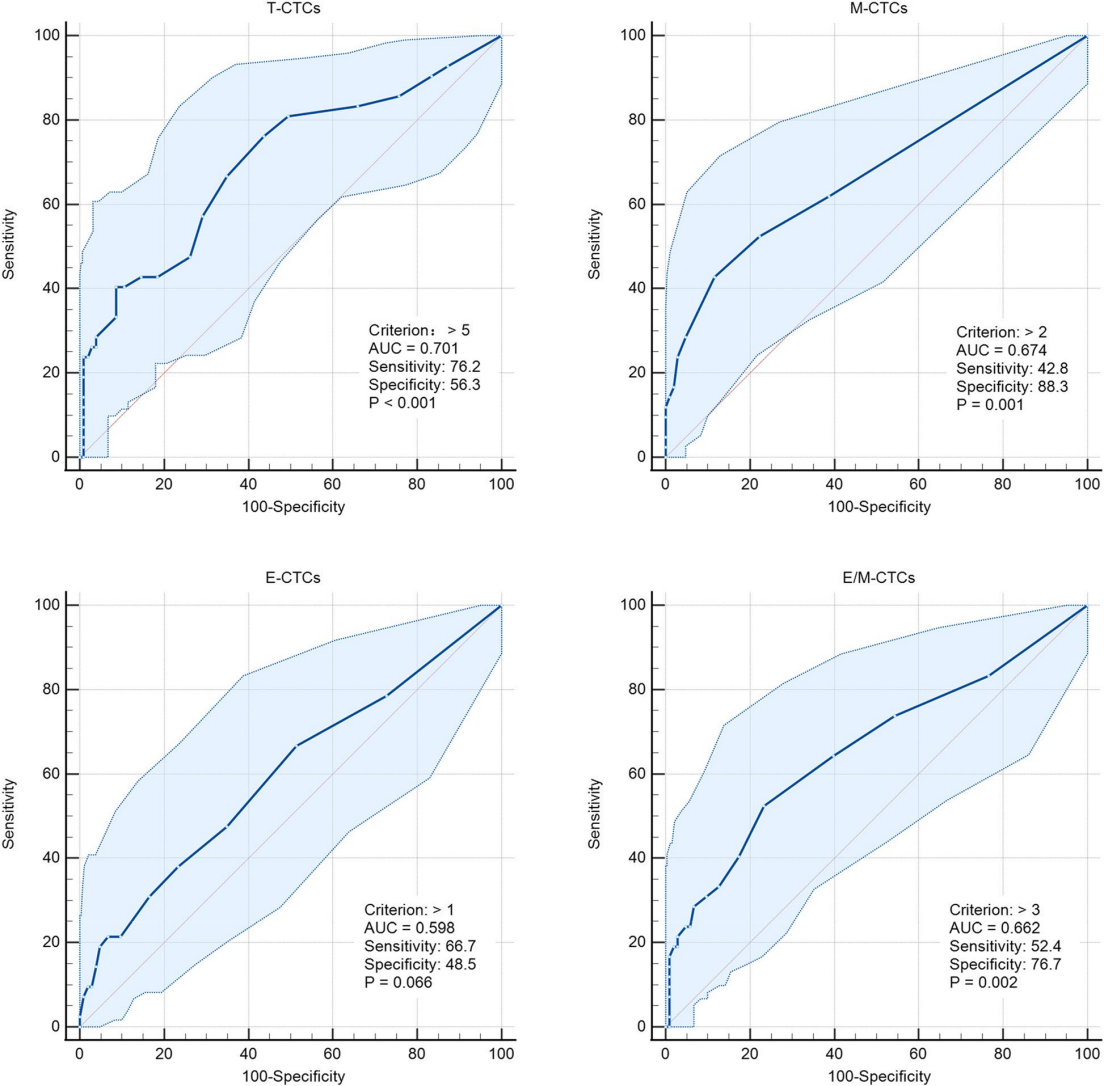
ROC curves of T-CTCs and subtypes to predict EPR

The Kaplan–Meier survival curve analysis showed that the OS of patients in the EPR group was significantly shorter than those in the EOR and LOR groups (*P* < 0.01), showing a significantly poor prognosis in the EPR group (Fig. 5). Therefore, the patients were divided into EPR and non-EPR groups to explore the risk factors for EPR. The ROC curve analysis indicated that the T-CTCs (0.701) exhibited the highest AUC to predict EPR as compared to those of M-CTCs (0.674), E-CTCs (0.598), and E/M-CTCs (0.662). The best cut-off value of T-CTCs was > 5/5 mL (Fig. 6).

### Analysis of risk factors for EPR

The factors, showing significance in univariate analysis, were included in the multivariate Cox proportional hazard risk analysis. However, the COX univariate analysis identified all the T-CTCs, M-CTCs, and E/M-CTCs as the risk factors for EPR. Considering the correlation between these indicators, the T-CTCs, which showed the best diagnostic EPR performance, were selected for univariate analysis. The results showed that T-CTCs > 5/5 mL (hazard ratio, HR = 2.417, 95% confidence interval (95% CI):1.143–5.111, *P* = 0.021), presence of MVI (HR = 2.471, 95% CI:1.002–6.091, *P* = 0.049), and presence of satellite nodules (HR = 2.105, 95% CI:1.046–4.239, *P* = 0.037) were independent risk factors for EPR (Table 2).

**Table 2:** Univariate and multivariate analyses to identify factors associated with the early progression recurrence of HCC

Characteristic	Comparison	Univariate			Multivariate		
		HR	95% CI	*P*-value	HR	95% CI	*P*-value
Sex	Male *vs.* Female	0.508	0.250–1.034	0.062			
Age, year	≥ 45 *vs.* < 45	0.621	0.339–1.138	0.123			
BMI, kg/m^2^	≥ 24 *vs.* < 24	0.304	0.135–0.686	0.004	0.575	0.236–1.406	0.225
Diabetes mellitus	Yes *vs.* No	0.494	0.119–2.046	0.331			
HBsAg	Positive *vs.* Negative	2.352	0.568–9.732	0.238			
HBV-DNA, 10^2^ IU/mL	≥ 5 *vs.* < 5	1.136	0.581–2.220	0.710			
Child-Pugh class	A stage *vs.* B stage	0.581	0.179–1.883	0.365			
TBil, μmol/L	≥ 17.1 *vs.* < 17.1	1.689	0.920–3.102	0.091			
ALB, mg/L	≥ 35 *vs.* < 35	0.605	0.268–1.364	0.225			
ALBI	> -2.60 *vs.* ≤ -2.60	1.450	0.777–2.703	0.243			
ALT, U/L	≥ 40 *vs.* < 40	0.618	0.328–1.162	0.135			
AST, U/L	≥ 40 *vs.* < 40	2.213	1.149–4.260	0.018	1.156	0.585–2.285	0.676
PT, s	≥ 13 *vs.* < 13	1.118	0.610–2.049	0.717			
INR	≥ 1 *vs.* < 1	1.032	0.507–2.099	0.931			
Platelet count, 10^9^ /L	≥ 225 *vs.* < 225	1.787	0.958–3.333	0.068			
AFP,μg/mL	≥ 200 *vs.* < 200	2.566	1.310–5.027	0.006	1.617	0.784–3.333	0.193
Tumor size, cm	≥ 5 *vs.* < 5	5.036	1.795–14.129	0.002	2.257	0.752–6.778	0.147
Multiple lesions	Yes *vs.* No	2.622	1.424–4.827	0.002	1.556	0.758–3.196	0.229
Inflow blood occlusion	Yes *vs.* No	1.368	0.654–2.860	0.405			
Tumor capsule	Complete *vs.* Incomplete	0.414	0.217–0.790	0.007	0.642	0.318–1.295	0.216
Resection margin, cm	≥ 1 *vs.* < 1	1.670	0.901–3.095	0.104			
MVI	Positive *vs.* Negative	5.143	2.272–11.640	0.000	2.471	1.002–6.091	0.049
Histological grade	≥ 3 *vs.* < 3	1.119	0.609–2.055	0.718			
Histological cirrhosis	Yes *vs.* No	0.810	0.431–1.523	0.513			
Satellite nodule	Yes *vs.* No	3.395	1.847–6.238	0.000	2.105	1.046–4.239	0.037
Ki67	≥ 35% *vs.* < 35%	2.769	1.478–5.186	0.001	1.695	0.850–3.382	0.134
Postoperative adjuvant therapy	Yes *vs.* no	0.783	0.419–1.462	0.442			
T-CTC count, n/5 mL	> 5 *vs.* ≤ 5	3.912	1.909–8.015	0.000	2.417	1.143–5.111	0.021
E-CTC count, n/5 mL	> 1 *vs.* ≤ 1	1.897	0.997–3.610	0.051			
M-CTC count, n/5 mL	> 2 *vs.* ≤ 2	4.397	2.362–8.183	0.000			
M/E-CTC count, n/5 mL	> 3 *vs.* ≤ 3	3.060	1.666–5.621	0.000			

*Abbreviations*: *AFP* Alpha-fetoprotein, *ALB* Albumin, *ALBI* Albumin-bilirubin, *ALT* Alanine aminotransferase, *AST* Aspartate aminotransferase, *BMI* Body mass index, *E-CTCs* Epithelial-circulating tumor cells, *HBV-DNA* Hepatitis B virus DNA, *HBsAg* Hepatitis B surface antigen, *HCC* Hepatocellular carcinoma, *INR* International normalized ratio, *M-CTCs* Mesenchymal-circulating tumor cells, *M/E-CTCs* Mesenchymal/epithelial-circulating tumor cells, *MVI* Microvascular invasion, *PT* Prothrombin time, *TBil* Total bilirubin, *T-CTCs* Total-circulating tumor cells

**Fig. 7:**
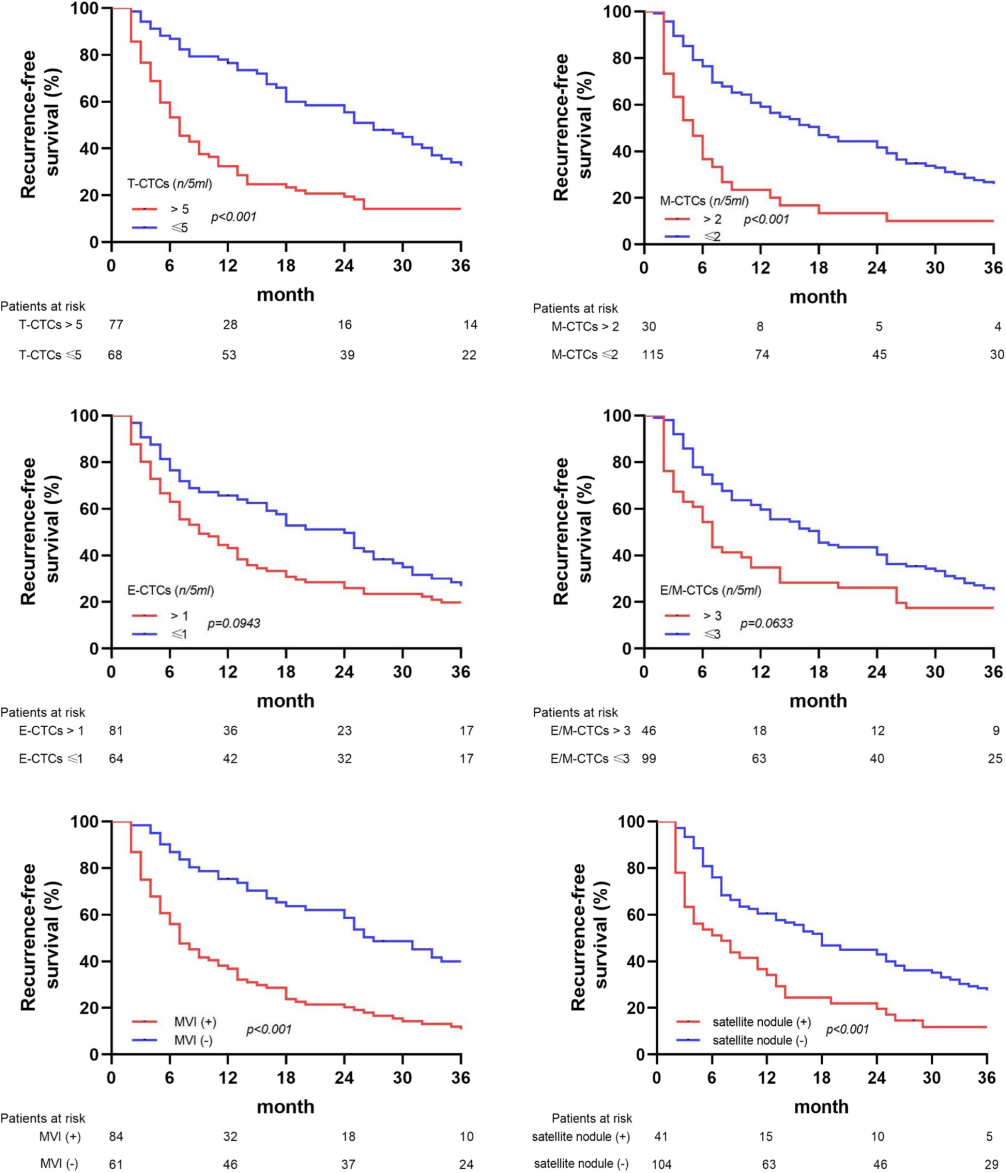
Kaplan–Meier survival curve analysis of RFS in HCC patients based on T-CTCs, subtypes of CTCs, MVI, and satellite nodules

Kaplan–Meier survival curve analysis showed that the patients with a higher CTCs (> 5/5 mL) and the presence of MVI and satellite nodules were more likely to have RFS as compared to those with lower CTCs or absence of MVI and satellite nodules (Fig. 7).

### Comparison of predictive potential of independent prognostic factors

**Fig. 8:**
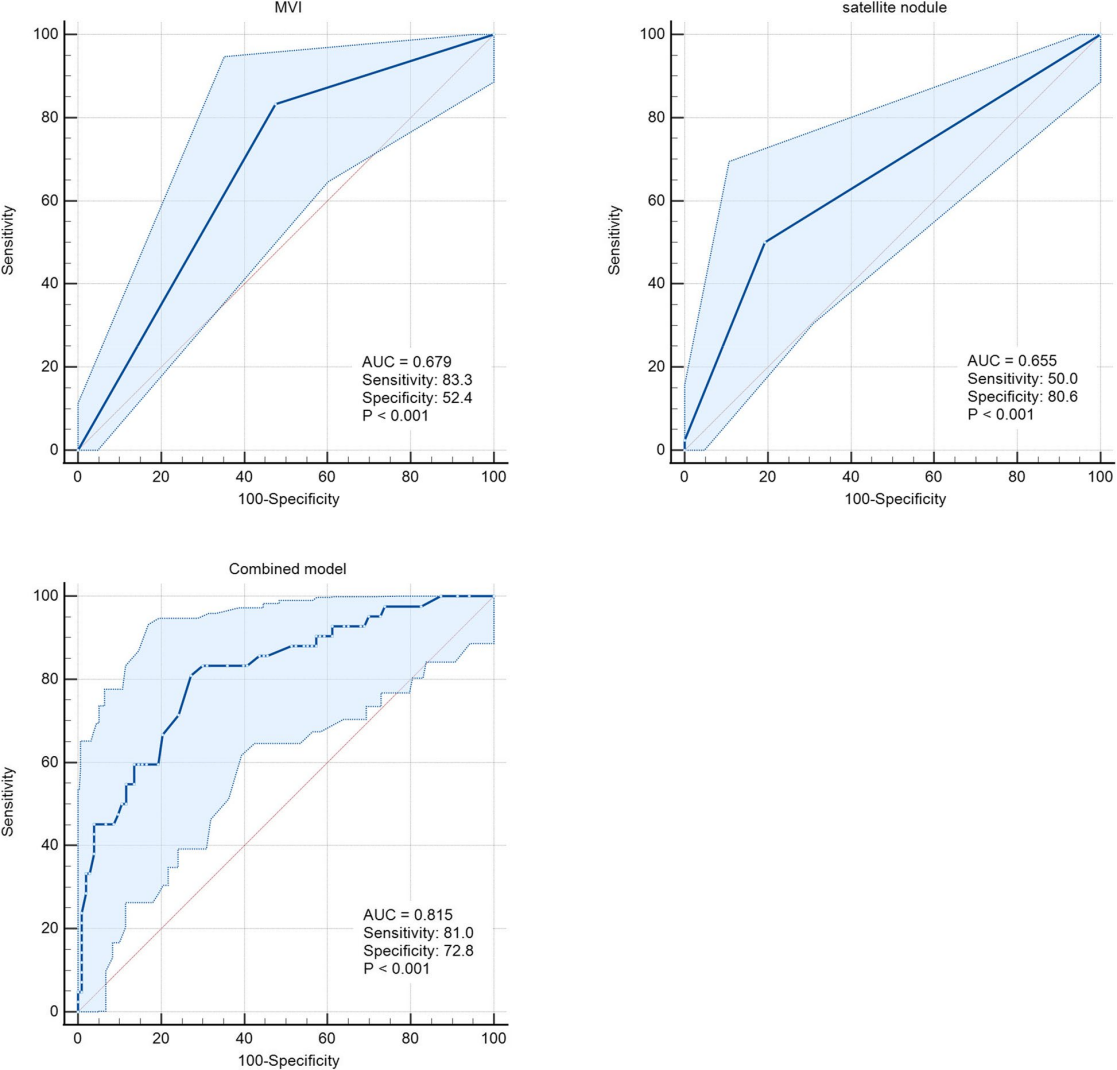
ROC curves of postoperative markers and their combined model to predict EPR

The ROC curve was used to compare the performance of risk factors to predict EPR separately and combined index model. The C-index of the combined three-index model (0.815) was higher than that of the single index model of T-CTCs (0.701), MVI (0.679), satellite nodules (0.655), and combined two-index models (Figs. 6 and 8). The prognostic performance of the combined model was better than those of the single-index or two-index models. The sensitivity and specificity of the combined model were 81.0 and 72.8, respectively.

## Discussion

HCC is a leading cause of cancer-related deaths worldwide [1, 25]. Hepatectomy is still the main radical treatment for HCC. Although the postoperative PR of HCC has been defined recently, numerous studies have mentioned the problem of refractory recurrence and fatal recurrence after the surgical removal of the tumor [26–28]. In the past, some studies have also focused on the effects of HCC recurrence patterns on treatment choice [10–12]. However, the classification mainly considers the treatment after recurrence, which has no clear effects on the OS of HCC patients with postoperative recurrence. The patient’s recurrence conditions have not been fully explored yet. Qi et al. [9] proposed a recurrence classification according to the conditions of patients using a multi-center and large sample of HCC patients with recurrence, which showed a crucial significance in guiding the treatment and predicting the survival of patients. Types III-IV are the types of PR, indicating the extrahepatic metastasis and intrahepatic dissemination of tumor. Most of the patients had refractory recurrences and were put together. The previous studies on recurrence have focused more on the time of recurrence and have shown that the recurrences within 2 years are correlated to the dissemination of primary tumor [6, 7, 21, 29]. The recurrences after 2 years are considered due to multi-centric origin [6, 30, 31]. Both the ER and PR of HCC are important factors, affecting the prognosis of HCC. Therefore, the pattern and time to recurrence must be considered comprehensively to analyze the prognosis of HCC recurrence. In this study, the results showed that the patients with EPR after hepatectomy had the worst survival prognosis. The patients with stage 0-B BCLC HCC, who could be treated with radical resection, might not show obvious invasion and metastasis [21, 22]. However, EPR also occurred in some patients. Therefore, exploring the risk factors of EPR is of great clinical significance.

The detection of CTCs in blood circulation was direct evidence of tumor metastasis [32]. HCC is a malignant tumor, and its main dissemination route is blood dissemination [6, 11]. With the improvements in CTCs separation and enrichment technology, CTCs can be stably detected [21, 22]. Using different methods, numerous studies have shown that CTCs were correlated with the poor prognosis of HCC [17, 22, 33, 34]. In this study, CTCs were also detected in the patients with BCLC stage 0-B and showed significant differences in different stages. These results were consistent with those of the previous studies [21, 22]. According to the process of epithelial-mesenchymal transition (EMT), the CTCs lose epithelial features and acquire mesenchymal features, which increase their metastatic potential and cause tumor dissemination before surgery, thereby promoting ER or PR [7, 35]. However, the presence of a small number of CTCs in blood might not lead to metastasis and dissemination. The shear force of blood flow and the monitoring effects of immune cells in the human body can eliminate CTCs and reduce tumor progression [36–38]. Moreover, some CTCs, released into circulation, might undergo apoptosis [39]; it has been shown in previous studies. The present study suggested that CTCs were detected in both the early and late HCC recurrence patients; however, there were significant differences in the numbers of T-CTCs, M-CTCs, E-CTCs, and E/M-CTCs between the patients with early and late HCC recurrence. Only T-CTCs had the predicted AUC value of ER > 0.7. The best cut-off value for predicting ER was T-CTCs > 4/5 mL. These results were similar to those of the previous studies [34, 40]. However, the current studies on the correlations between PR and CTCs are limited. Some studies on postoperative metastasis have also shown differences in CTCs [41, 42]. However, only a previous study by Qi et al. [9] showed that there was a significant difference in the number of CTCs between the HCC patients having PR and non-PR. The current study showed that, except E-CTCs, there were significant differences in the numbers of the other T-CTCs, including the highly aggressive M-CTCs and E/M-CTCs, between the PR and OR groups. This indicated that HCC patients with PR have more aggressive CTCs. The median counts of T-CTCs, M-CTCs, and E/M-CTCs to predict PR were > 12/5 mL, > 2/5 mL, and > 3/5 mL, respectively.

According to the recurrence times and patterns, the patients were re-divided into four groups, including EOR, EPR, LOR, and LPR. LPR occurred in only one patient, which was consistent with the results of the previous studies [6, 7, 11, 29, 30]. The dissemination and metastasis of tumor after 2 years might be due to the recurrence of tumor. However, routine postoperative follow-up can early detect recurrent tumors. Kaplan–Meier survival curve analysis found that the patients with EPR had significantly shorter OS as compared to those in the other two groups, and most patients died within 2 years. A few HCC patients with single lung metastasis or intraperitoneal implantation achieved long-term survival with further radical treatment. Although the patients enrolled in this study had early- and middle-stage HCC, the OS of patients with EPR did not reach the median survival time of all HCC patients according to epidemiological statistics [25, 43]. Therefore, it was speculated that most patients with EPR might not get survival benefits from surgery. However, the EPR of HCC has not been investigated in previous studies. Further investigation suggested that the counts of T-CTCs, M-CTCs, and E/M-CTCs were significantly higher in the EPR group as compared to those in the EOR and LOR groups. However, there were significant differences in only M-CTC count between the EOR and LOR groups, while the counts of T-CTCs, E-CTCs, and E/M-CTCs showed no significant differences. Therefore, it was suggested that EPR was correlated with CTCs and might be caused by the early metastasis and dissemination of HCC, which might not completely cause EOR. The AUC of T-CTCs to predict EPR was 0.701, which was the largest among all the AUCs. The optimal cut-off value was > 5/5 mL, which was not significantly different from the diagnosis of ER. In order to further explore the clinical factors, affecting the occurrence of EPR, the clinical factors, which might be correlated to recurrence, were selected for COX regression analysis. In addition to CTCs, the presence of MVI and satellite nodules was also taken as risk factors for EPR. Both the MVI and satellite nodules are evidence of HCC invasion and metastasis [6, 44, 45]. Numerous previous studies as well as the current study showed that the MVI and satellite nodules play an important role in HCC recurrence [44–47]. However, the presence of MVI and satellite nodules was not as effective as CTCs in predicting EPR and could only be accurately obtained by surgery. The preoperative clinical indicators included in this study were not correlated with EPR. The current study showed that EPR was correlated with CTCs preoperatively. Therefore, the only way to accurately predict EPR before surgery might be through CTCs detection. The combined model of T-CTCs, MVI, and satellite nodules showed optimal diagnostic efficiency and might have a certain role in guiding postoperative adjuvant therapy. Although the study did not show positive results for adjuvant therapy to improve the form of postoperative recurrence, this needs to be further explored by expanding the sample size.

The new HCC recurrence model could not fully predict the prognosis of HCC patients. However, its combination with recurrence time predicted a group of patients with the worst postoperative survival. As a method for preoperative detection of tumor metastasis and dissemination, CTCs might have an important role in predicting ER, PR, and EPR of HCC after hepatectomy. Therefore, future studies should focus more on exploring adjuvant therapies, which might effectively reduce CTCs in peripheral blood, or exploring effective treatments under different CTCs counts to prevent EPR and prolong the OS of HCC patients.

This study had several limitations. First, in the process of CTC aggregation, some small tumor cells might filter through the membrane, resulting in false negative results. Second, the release of CTCs during surgery was not evaluated in this study. Third. This study was a single-center study with small sample size. The patients with LPR occurred less frequently during follow-up, and its comparison with EPR was not further analyzed.

In conclusion, the HCC patients with EPR had the worst prognosis. CTCs analysis before surgery could predict the ER, PR, and EPR of HCC. The preoperative T-CTC count had a higher predictive efficiency as compared to the other CTC subtypes, and the best cut-off value to predict postoperative EPR was > 5/5 mL. However, the combined model of T-CTCs, MVI, and satellite nodules showed significantly better performance as compared to the single-index or two-index models. The AUC, sensitivity, and specificity of the combined model were 0.815, 81.0%, and 72.8%, respectively.

### Supplementary Information

**Additional file 1: Figure S1.** Distribution of T-CTCs and subtypes in the patients with HCC BCLC 0-A stage and B stage.

**Additional file 2: Figure S2.** ROC curves of T-CTCs and subtypes to predict ER as compared to LR.

**Additional file 3.**

## Data Availability

The data used and analysed during the current study available from the corresponding author on reasonable request.

## References

[1] Bertuccio P, Turati F, Carioli G (2017). Global trends and predictions in hepatocellular carcinoma mortality. J Hepatol.

[2] Glantzounis GK, Korkolis D, Sotiropoulos GC (2022). Individualized approach in the surgical management of hepatocellular carcinoma: results from a Greek multicentre study. Cancers (Basel).

[3] Orimo T, Kamiyama T, Kakisaka T (2022). Hepatectomy is beneficial in select patients with multiple hepatocellular carcinomas. Ann Surg Oncol.

[4] Fukami Y, Kaneoka Y, Maeda A (2020). Liver resection for multiple hepatocellular carcinomas: a Japanese nationwide survey. Ann Surg.

[5] Bruix J, Gores GJ, Mazzaferro V (2014). Hepatocellular carcinoma: clinical frontiers and perspectives. Gut.

[6] Heimbach JK, Kulik LM, Finn RS (2018). AASLD guidelines for the treatment of hepatocellular carcinoma. Hepatology.

[7] Kobayashi T, Aikata H, Kobayashi T (2017). Patients with early recurrence of hepatocellular carcinoma have poor prognosis. Hepatobiliary Pancreat Dis Int.

[8] Ivanics T, Murillo Perez CF, Claasen M (2022). Dynamic risk profiling of HCC recurrence after curative intent liver resection. Hepatology.

[9] Qi LN, Ma L, Wu FX (2022). Clinical implications and biological features of a novel postoperative recurrent HCC classification: a multi-centre study. Liver Int.

[10] Tabrizian P, Jibara G, Shrager B (2015). Recurrence of hepatocellular cancer after resection: patterns, treatments, and prognosis. Ann Surg.

[11] Wen T, Jin C, Facciorusso A (2018). Multidisciplinary management of recurrent and metastatic hepatocellular carcinoma after resection: an international expert consensus. Hepatobiliary Surg Nutr.

[12] Xu XF, Xing H, Han J (2019). Risk factors, patterns, and outcomes of late recurrence after liver resection for hepatocellular carcinoma: a multicenter study from China. JAMA Surg.

[13] Mise Y, Hasegawa K, Shindoh J (2015). The feasibility of third or more repeat hepatectomy for recurrent hepatocellular carcinoma. Ann Surg.

[14] Yamashita Y, Shirabe K, Tsuijita E (2013). Third or more repeat hepatectomy for recurrent hepatocellular carcinoma. Surgery.

[15] Wong PC, She WH, Ma KW (2022). Impact of time to recurrence on survival outcome of salvage liver transplantation. J Gastrointest Surg.

[16] Jerabkova-Roda K, Dupas A, Osmani N (2022). Circulating extracellular vesicles and tumor cells: sticky partners in metastasis. Trends Cancer.

[17] Qi LN, Xiang BD, Wu FX (2018). Circulating tumor cells undergoing EMT provide a metric for diagnosis and prognosis of patients with hepatocellular carcinoma. Cancer Res.

[18] Markou A, Tzanikou E, Lianidou E (2022). The potential of liquid biopsy in the management of cancer patients. Semin Cancer Biol.

[19] Xie X, Li Y, Lian S (2022). Cancer metastasis chemoprevention prevents circulating tumour cells from germination. Signal Transduct Target Ther.

[20] Wang Z, Luo L, Cheng Y (2018). Correlation between postoperative early recurrence of hepatocellular carcinoma and mesenchymal circulating tumor cells in peripheral blood. J Gastrointest Surg.

[21] Chen VL, Xu D, Wicha MS (2020). Utility of liquid biopsy analysis in detection of hepatocellular carcinoma, determination of prognosis, and disease monitoring: a systematic review. Clin Gastroenterol Hepatol.

[22] Ahn JC, Teng PC, Chen PJ (2021). Detection of circulating tumor cells and their implications as a biomarker for diagnosis, prognostication, and therapeutic monitoring in hepatocellular carcinoma. Hepatology.

[23] Reig M, Forner A, Rimola J (2022). BCLC strategy for prognosis prediction and treatment recommendation: the 2022 update. J Hepatol.

[24] Edmondson HA, Steiner PE (1954). Primary carcinoma of the liver: a study of 100 cases among 48,900 necropsies. Cancer.

[25] Sung H, Ferlay J, Siegel RL (2021). Global cancer statistics 2020: GLOBOCAN estimates of incidence and mortality worldwide for 36 cancers in 185 countries. CA Cancer J Clin.

[26] Kemeny HR, Elsamadicy AA, Farber SH (2020). Targeting PD-L1 initiates effective antitumor immunity in a murine model of Cushing disease. Clin Cancer Res.

[27] Kim BW, Kim YB, Wang HJ (2006). Risk factors for immediate post-operative fatal recurrence after curative resection of hepatocellular carcinoma. World J Gastroenterol.

[28] Sumie S, Nakashima O, Okuda K (2014). The significance of classifying microvascular invasion in patients with hepatocellular carcinoma. Ann Surg Oncol.

[29] Orrapin S, Udomruk S, Lapisatepun W (2022). Clinical implication of circulating tumor cells expressing epithelial mesenchymal transition (EMT) and cancer stem cell (CSC) markers and their perspective in HCC: a systematic review. Cancers (Basel).

[30] Lee KF, Chong CCN, Fong AKW (2018). Pattern of disease recurrence and its implications for postoperative surveillance after curative hepatectomy for hepatocellular carcinoma: experience from a single center. Hepatobiliary Surg Nutr.

[31] Imamura H, Matsuyama Y, Tanaka E (2003). Risk factors contributing to early and late phase intrahepatic recurrence of hepatocellular carcinoma after hepatectomy. J Hepatol.

[32] Cortés-Hernández LE, Eslami SZ, Alix-Panabières C (2020). Circulating tumor cell as the functional aspect of liquid biopsy to understand the metastatic cascade in solid cancer. Mol Aspects Med.

[33] Guo W, Sun YF, Shen MN (2018). Circulating tumor cells with stem-like phenotypes for diagnosis, prognosis, and therapeutic response evaluation in hepatocellular carcinoma. Clin Cancer Res.

[34] Zhang Q, Rong Y, Yi K (2020). Circulating tumor cells in hepatocellular carcinoma: single-cell based analysis, preclinical models, and clinical applications. Theranostics.

[35] Książkiewicz M, Markiewicz A, Zaczek AJ (2012). Epithelial-mesenchymal transition: a hallmark in metastasis formation linking circulating tumor cells and cancer stem cells. Pathobiology.

[36] Wirtz D, Konstantopoulos K, Searson PC (2011). The physics of cancer: the role of physical interactions and mechanical forces in metastasis. Nat Rev Cancer.

[37] Anvari S, Osei E, Maftoon N (2021). Interactions of platelets with circulating tumor cells contribute to cancer metastasis. Sci Rep.

[38] Kolostova K, Pospisilova E, Matkowski R (2022). Immune activation of the monocyte-derived dendritic cells using patients own circulating tumor cells. Cancer Immunol Immunother.

[39] Zhang Y, Li J, Cao L (2012). Circulating tumor cells in hepatocellular carcinoma: detection techniques, clinical implications, and future perspectives. Semin Oncol.

[40] Wang PX, Xu Y, Sun YF (2021). Detection of circulating tumour cells enables early recurrence prediction in hepatocellular carcinoma patients undergoing liver transplantation. Liver Int.

[41] Pan Y, Li D, Yang J (2021). Portal venous circulating tumor cells undergoing epithelial-mesenchymal transition exhibit distinct clinical significance in pancreatic ductal adenocarcinoma. Front Oncol.

[42] Li Z, Xu K, Tartarone A (2021). Circulating tumor cells can predict the prognosis of patients with non-small cell lung cancer after resection: a retrospective study. Transl Lung Cancer Res.

[43] Tandon P, Garcia-Tsao G (2009). Prognostic indicators in hepatocellular carcinoma: a systematic review of 72 studies. Liver Int.

[44] Chen ZH, Zhang XP, Feng JK (2022). Patterns, treatments, and prognosis of tumor recurrence after resection for hepatocellular carcinoma with microvascular invasion: a multicenter study from China. HPB (Oxford).

[45] Hu J, Zhang ZQ, Zhu W (2020). Comparison of clinicopathological traits and prognostic factors of hepatocellular carcinoma with and without cirrhotic background. Carcinogenesis.

[46] An C, Kim DW, Park YN (2015). Single hepatocellular carcinoma: preoperative MR imaging to predict early recurrence after curative resection. Radiology.

[47] Lee S, Kang TW, Song KD (2021). Effect of microvascular invasion risk on early recurrence of hepatocellular carcinoma after surgery and radiofrequency ablation. Ann Surg.

