# Supplementary figures and images for: Prognostic potential of preoperative circulating tumor cells to predict the early progression recurrence in hepatocellular carcinoma patients after hepatectomy
Source: BMC Cancer. 2023 Nov 27;23:1150. doi: 10.1186/s12885-023-11629-0 (PMC10680336; doi:10.1186/s12885-023-11629-0)

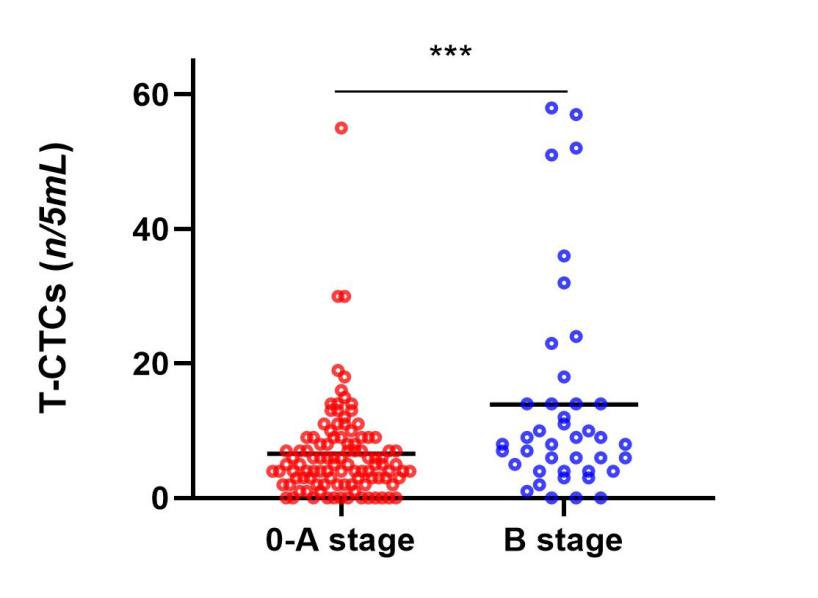

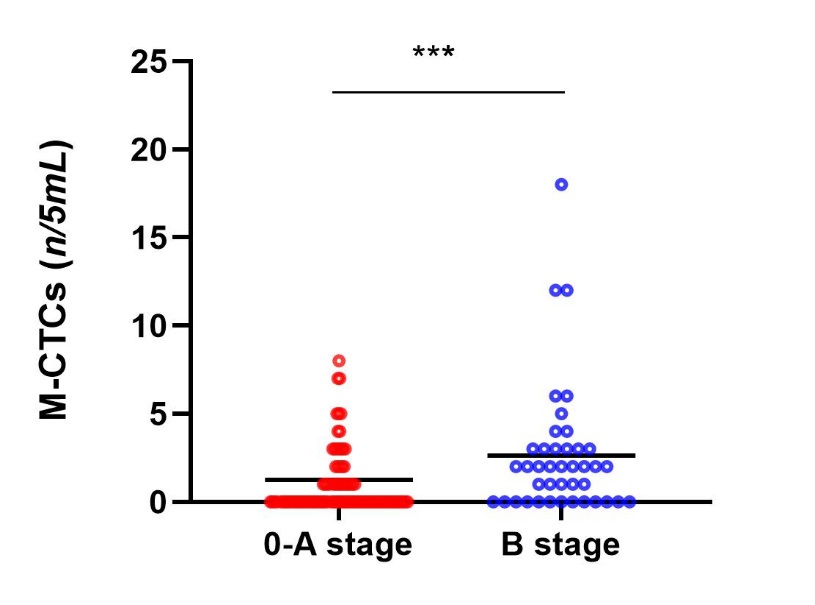


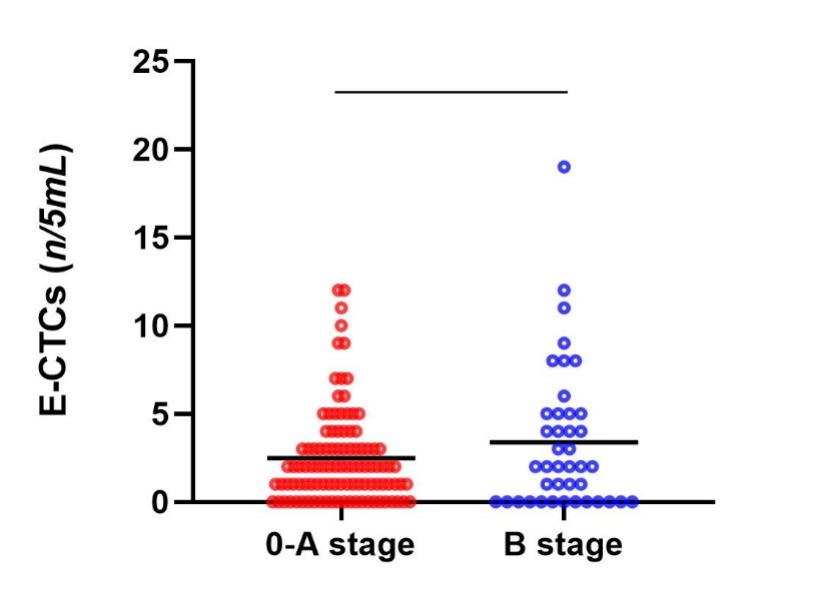

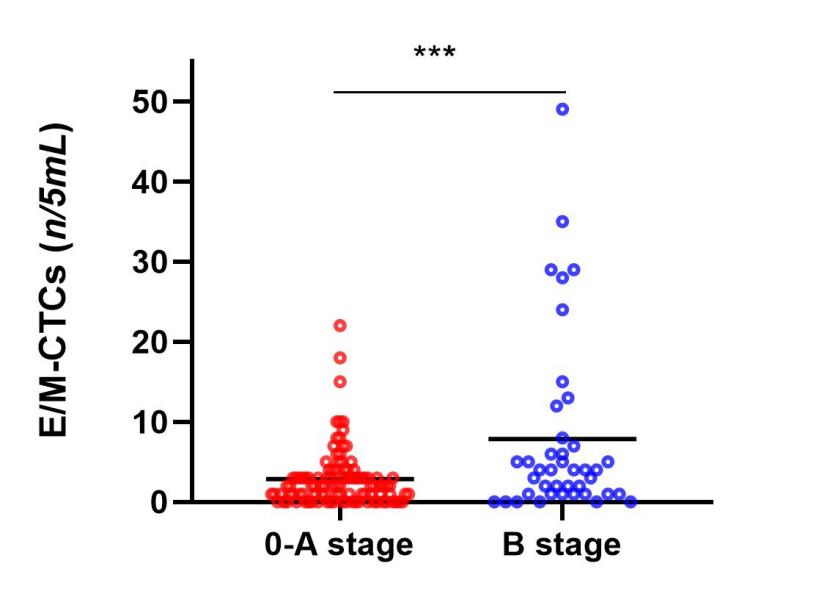


Figure S1. Distribution of T-CTCs and subtypes in the patients with HCC BCLC 0-A stage and B stage.

Supplement: Supplementary file 1 — Additional file 1: Figure S1. Distribution of T-CTCs and subtypes in the patients with HCC BCLC 0-A stage and B stage. [file 12885_2023_11629_MOESM1_ESM.docx]

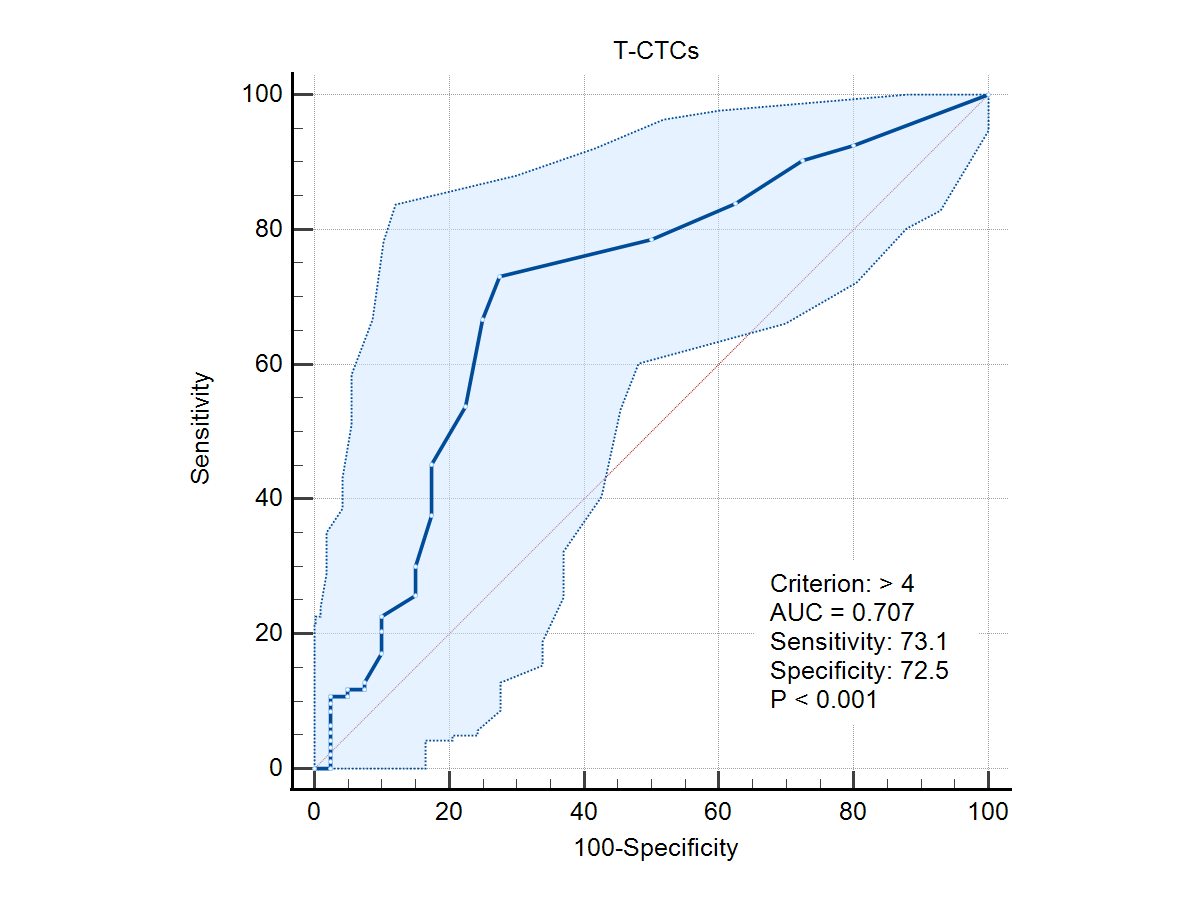

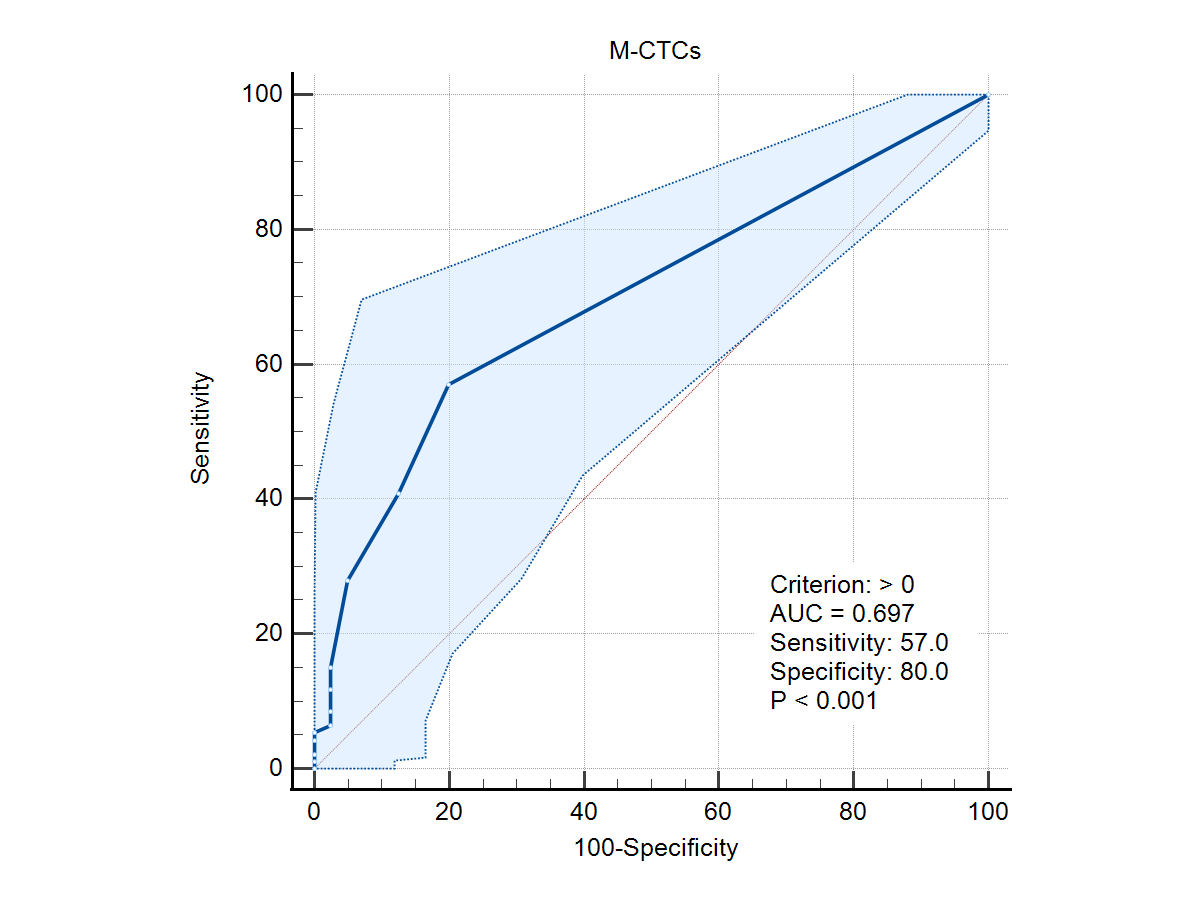


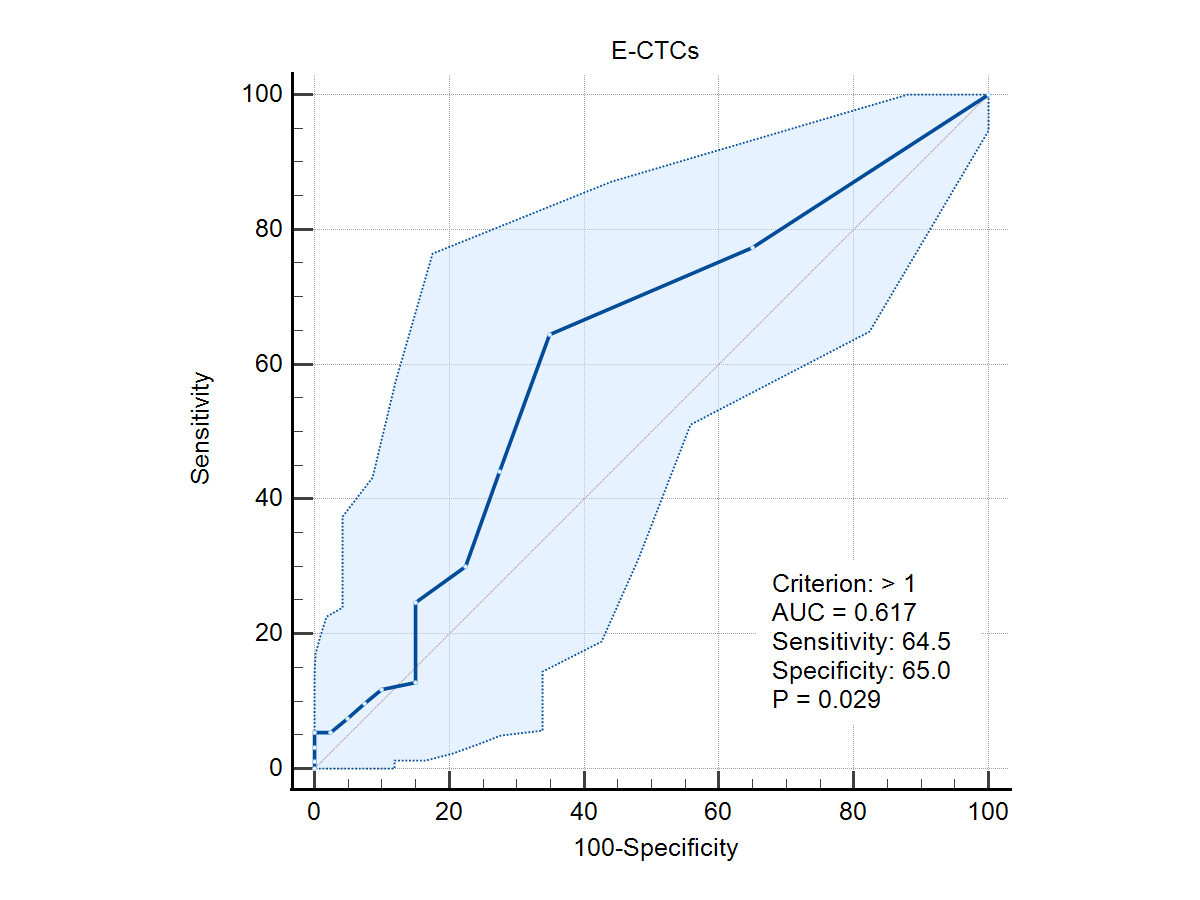

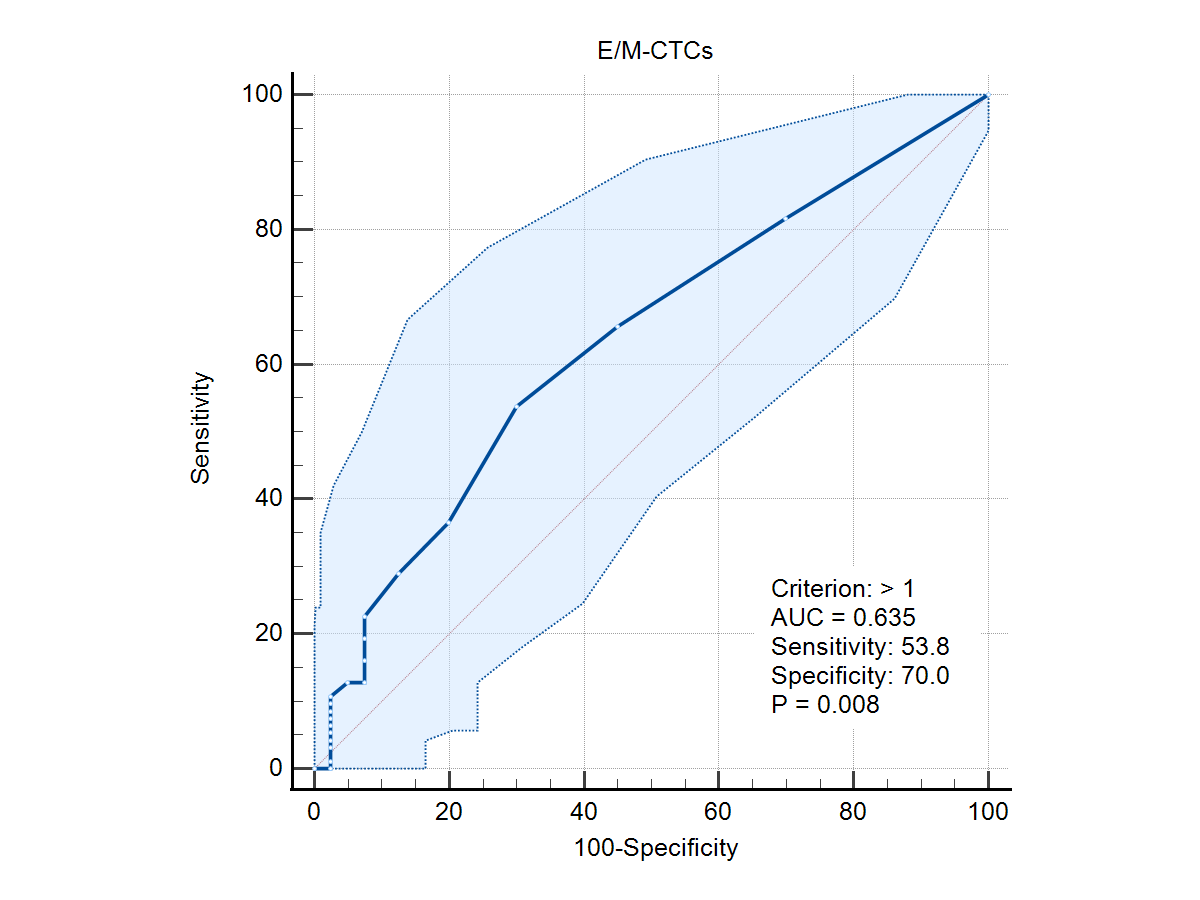


Figure S2. ROC curves of T-CTCs and subtypes to predict ER as compared to LR.

Supplement: Supplementary file 2 — Additional file 2: Figure S2. ROC curves of T-CTCs and subtypes to predict ER as compared to LR. [file 12885_2023_11629_MOESM2_ESM.docx]
